# Effects of an L-arginine-based multi ingredient product on endothelial function in subjects with mild to moderate hypertension and hyperhomocysteinemia - a randomized, double-blind, placebo-controlled, cross-over trial

**DOI:** 10.1186/s12906-017-1603-9

**Published:** 2017-02-02

**Authors:** Claudia A. Reule, Birgit Goyvaerts, Christiane Schoen

**Affiliations:** 1 Nutritional CRO, BioTeSys GmbH, Schelztorstraße 54-56, 73728 Esslingen, Germany; 2 Dr. Loges & Co. GmbH, Schützenstraße 5, 21423 Winsen, Luhe Germany

**Keywords:** Endothelial function, Hyperhomocysteinemia, Hypertension, Pycnogenol®, L-arginine, Folic acid

## Abstract

**Background:**

Nutrition plays an important role in prevention and management of cardiovascular diseases (CVD) in early stages. Recent research demonstrated beneficial effects of various nutritional ingredients on vascular health. The aim of the current study was to evaluate the effects of an L-arginine-based multi ingredient product (AbMIP) on vascular function.

**Methods:**

Twenty-five male and female subjects, aged between 45 and 65 years with elevated blood pressure and hyperhomocysteinemia were included in this cross-over trial. Subjects were randomly assigned to one of the two sequence groups (AbMIP -placebo or placebo – AbMIP). AbMIP and placebo were taken for 4 weeks, each. Endothelial function under fasting conditions, blood pressure, postprandial endothelial function after consumption of a high fat meal, homocysteine, asymmetric dimethyl arginine (ADMA) and Hba1c were determined.

**Results:**

AbMIP significantly improved fasting endothelial function determined by EndoPAT™ when compared to placebo (*p* = 0.047). Similarly, homocysteine levels were significantly decreased after verum supplementation when compared to placebo (*p* < 0.0001). Systolic blood pressure decreased significantly under AbMIP (*p* = 0.002) and the reduction was more pronounced when compared to placebo. However, due to placebo-effects no significant difference could be found between groups (*p* = 0.586). The effects on postprandial endothelial function were stronger for AbMIP when compared with placebo but did not reach significance (*p* = 0.201). No significant effects of AbMIP were observed regarding HbA1c, ADMA and diastolic blood pressure.

**Conclusions:**

Due to improvement on endothelial function, decrease of elevated homocysteine levels and excellent tolerability, AbMIP was demonstrated to be a beneficial option for dietary treatment of endothelial dysfunction and hyperhomocysteinemia in early stages of CVD.

**Trial registration:**

The clinical trials.gov identifier is NCT02392767, November 14, 2014.

## Background

Cardiovascular diseases (CVDs) are still the main causes of death worldwide. It is estimated, that 17 million subjects died from CVDs in 2012. The World health Organisation (WHO) stresses the need for early detection and therapy in subjects with CVD or at high cardiovascular risk (due to the presence of one or more risk factors such as hypertension, diabetes, hyperlipidaemia, overweight or already established disease) [1].

Endothelial dysfunction is regarded as an additional independent risk factor for CVD, which develops in early stages of atherosclerosis. In combination with other risk factors, like moderate hypertension or hyperhomocysteinemia, endothelial dysfunction indicates early stages of vascular changes [2, 3]. Besides inadequate activity behaviour, dietary habits of the western world contribute considerably to the development of CVDs considerably. Larger and more frequent meals are leading to longer postprandial states, which are regarded as a critical factor for the development of atherogenesis [4]. Considering the recommendation of the WHO for early health management after detection of elevated risk for CVDs, attention should be drawn to the role of nutrition in the maintenance of vascular health. Several nutritional ingredients have been shown to be able to modulate or improve different physiologic functions or conditions. Ingredients with high anti-oxidative or anti-inflammatory properties, like cocoa, lycopene or pycnogenol® showed beneficial effects on endothelial function or postprandial endothelial function [5–10]. Another ingredient, which is supposed to have a positive influence on vascular function, is L-arginine, a semi essential amino acid, which is the substrate for nitric oxide synthase (NO-synthase) [11, 12]. NO, the endothelium-derived relaxing factor, is responsible for endothelium dependent vasodilation [13]. The enzyme NO-synthase oxidizes L-arginine to NO in the endothelium as needed. NO then diffuses to the smooth muscle cell layer, causing muscle relaxation and therefore leading to vasodilation. Further studies have shown positive effects of L-arginine on postprandial endothelial function in a healthy collective [14]. Effects of L-arginine in hypertensive disorder of pregnancy and in peripheral arterial diseases have been observed, but results were inconsistent and some observations lacked significant effects [15–17]. Another known risk factor for CVDs, is hyperhomocysteinemia. Homocysteine is a sulfur-containing intermediate product in the normal metabolism of methionine, with potentially cell-toxic properties [18]. Levels above 10 μmol/l increase the risk for CVDs. Folic acid deficiency is considered to be the most common cause of hyperhomocysteinemia [18]. Folic acid, vitamin B12 and B6 are able to decrease elevated homocysteine levels reliably [18–21].

As described above, various nutritional ingredients have the potential to influence different risk factors for CVDs. The aim of the following study was to evaluate the influence of a preparation containing several health-supporting ingredients for endothelial function, blood pressure, homocysteine and other biomarkers.

## Methods

### Study design

The study was performed as monocentric, randomized, placebo-controlled, double-blind cross-over study at the study site BioTeSys GmbH, Esslingen, Germany between October 2014 and August 2015. The study was conducted in accordance with the declaration of Helsinki, complied with principles of Good Clinical Practice (ICH-GCP) and was approved by the responsible Institutional Review Board (Ethics Committee Landesaerztekammer Baden-Wuerttemberg).

After signing the informed consent and prior to trial start, subjects were screened for eligibility. Documentation of demographic data, medical history, nutrition status, vital signs (ECG and blood pressure) and routine blood parameters, including prothrombin time (PT), were recorded. Additionally, homocysteine levels were determined. Subsequently, subjects were instructed about blood pressure reading at home, on the left arm, after at least 10 min of rest, in a sitting position. All subjects received the same blood pressure device (boso medical, PC2) and the 7-day blood pressure diary to measure their blood pressure during the 7 days before the study start at home.

All subjects eligible at visit 1 were randomized to one of the two sequence groups and the study procedures were performed.

At visit 1 adverse events, concomitant medication, endothelial function and routine blood parameters including PT were determined. At the end of the visit, the subjects received study preparations for a period of 4 weeks. At the end of the first supplementation phase (visit 2), endothelial function, postprandial endothelial function, homocysteine, adverse events, concomitant medication, compliance, tolerability, asymmetric dimethyl arginine (ADMA), HbA1c, blood pressure, and routine blood parameters including PT were determined, followed by a wash out period of 8 weeks. For the second supplementation phase, visit 3 and visit 4 were performed following the same procedures performed at visits 1 and 2. Before each visit, subjects measured and documented their blood pressure daily for a period of 7 days. For detailed description see the particular method section.

### Intervention

During supplementation phases AbMIP (vasoLoges® protect, Dr. Loges & Co. GmbH, Germany) or placebo was taken by subjects for 4 weeks, each, in randomized order. Two tablets had to be taken twice daily together with a meal and sufficient liquid. Daily dose of AbMIP contains 2400 mg L-arginine, 80 mg Pycnogenol®, 45 μg vitamin K2, 10 mg alpha lipoic acid, 8 mg vitamin B6, 500 μg vitamin B12 and 600 μg folic acid. The placebo corresponded to the verum in taste, smell and appearance but did not contain the active ingredients. These were replaced by maize starch. The study preparations were randomly assigned to the subjects (allocation ratio 1:1), according to the randomisation scheme generated by the sponsor (Dr. Loges & Co. GmbH, Germany), using the software “Multizufall” with block size 2 and stratified by gender. Double-blind product assignment was ensured by labelled study preparations handed out by the investigator. Besides the subjects, all parties involved in the study performance were blinded until completion of the study and unlocking of the data base.

### Subjects

Eighteen male and 7 female subjects were included in the current trial. The following inclusion criteria had to be fulfilled: Postmenopausal women and men, non-smoking, between 40 and 65 years old, BMI between 20 and 32 kg/m^2^, homocysteine level above or equal to 10 μmol/l, elevated systolic blood pressure (mean of 130–149 mmHg measured during a period of 7 days before visit 1) but without requiring medical therapy. Exclusion criteria were: Sleep apnoea, CVDs requiring medical therapy, further chronic disease, like diabetes mellitus, malignant disease, kidney disease, liver disease or psychosis. Furthermore, subjects taking any medication or food supplements that could interfere with the study procedures, like statins, blood pressure medication, vitamin B, folic acid, L-arginine, Pycnogenol® etc., were excluded. Subjects with known HIV, hepatitis B or C infection, drug or alcohol addiction were not included in this trial.

### Nutritional and physical recommendations

Subjects were asked not to change their nutritional habits or sporting activities during the study period. At the end of each supplementation phase, possible changes in nutritional habits, sporting activities or weight were documented for control. Consumption of alcohol was not allowed 48 h prior to and during the visits. The meal before each visit was standardized for all subjects (bread with low-fat cream cheese and tomatoes or cucumber). Twenty-four hours before each visit, the subjects had to avoid sport or exhaustive activities.

### Outcome measures

#### Endothelial function

As primary objective the change of fasting endothelial function before and after supplementation compared against placebo was defined. Endothelial function was determined, using EndoPAT™ 2000 (Itamar medical Ltd, Caesarea, Israel) (non-invasive Peripheral Aterial Tonometry) using a reactive hyperemia procedure. The outcome measure was the change in endothelial function during the intervention period of 4 weeks. The endothelial function was determined as the natural log of the Reactive Hyperemia Index (“lnRHI”), which is the post-to-pre occlusion peripheral arterial tonometry signal ratio in the occluded side, relative to the same ratio in the control side, corrected for baseline vascular tone of the occluded side. Normal lnRHI > 0.51, Abnormal lnRHI < 0.51.

LnRHI was determined at each visit after overnight fasting, after at least 20 min acclimatization at study site and 10 min rest in a lying position before start of measurement.

For each volunteer, the measurement took place at the same time of day for each visit under standardized conditions (silent room without harsh light and constant room temperature between 21 and 24 °C). The test is performed at the fingertip. After assessing a baseline signal for 5.5 min, blood flow was occluded at the non dominant upper-arm for 5 min. After ischemia the endothelial-dependent dilatation of blood vessels was measured for 5 min. Data prior to and after ischemia was analyzed automatically by integrated EndoPAT™ 2000 Software (3.5.4).

As secondary parameter, the postprandial endothelial function was determined after both supplementation phases. Following the above described measurement of fasting endothelial function, the subjects received a high fat meal consisting of 200 ml cream with 30% fat content. Sixty minutes after intake of high fat meal, a second endothelial function measurement was performed. The difference between the fasting endothelial function and the endothelial function after high fat meal was defined as postprandial reaction of endothelial function.

#### Blood pressure

The blood pressure was measured daily by subjects at home as described above, starting 7 days before each visit. The mean of systolic and diastolic blood pressure measured within these 7 days was used for analysis.

#### Homocysteine

Homocysteine levels were determined batchwise in serum using high performance liquid chromatography at the end of the study. In order to ensure sample stability, serum samples were centrifuged exactly 30 min after blood sampling, transferred into aliquots and stored below −70 °C.

#### Other biomarkers

At the end of each supplementation phase, ADMA and HbA1c were determined. Serum samples for ADMA analysis were handled as described above and analyzed batchwise using an enzymatic test (Immundiagnostik AG, Germany). HbA1c was determined in ethylenediaminetetraacetate (EDTA) stabilized blood using high performance liquid chromatography.

#### Safety assessments

To assess the safety of the study products, blood samples for routine laboratory, including haemogram (complete blood count), liver enzymes, blood lipids, blood glucose, uric acid, creatinine and PT, were taken at each visit. Vital signs were determined at all visits. Additionally, adverse events and concomitant medication were documented. The tolerability of the study products was rated by the subjects at the end of each supplementation phase, by selecting one of the following categories: “well tolerated”, “slightly unpleasant” or “very unpleasant”.

### Statistics

#### Sample size calculation

For sample size calculation an intervention effect of RHI = 0.3 with a standard deviation of 0.45 in comparison to placebo was assumed. A priori sample size calculation was performed, using the software g*power 3.1.2. with the following input details: Two tailed, effect size d = 0.7, Alpha error problem: α = 0.05, actual power: 80%, correlation between groups: 0.5. Based on the calculations, the study should be performed with 20 subjects. Considering a drop-out rate of 20%, the study should be performed with 25 subjects. This sample size was also confirmed by Enseleit et al. [8]. In this study the influence of Pycnogenol® on endothelial function in 23 subjects was evaluated. A comparable study design was used by Grassi et al. to evaluate the influence of cocoa on endothelial function, which was performed with 20 subjects using a cross-over design [8].

#### Statistical analysis

All statistical tests were performed two-sided, using SPSS Version 17.0 and GraphPad Prism Version 5.04. Significance level was set to 5%. The distribution of parameters was tested for normality using the Shapiro-Wilk test for each single parameter which was the basis for selecting parametric or non-parametric approach (non-normality distribution with alpha level 0.1). Before analysis, carry-over and period effects as well as baseline differences were checked for all parameters. No issues were found and consequently all parameters could be analyzed using the statistical test procedure for cross-over trials [22]. For evaluation of product effects the intra-individual differences between the two supplementation phases were analyzed using unpaired test statistics (unpaired *t*-test or Mann–Whitney *U* test) between the sequence groups (verum-placebo/placebo-verum). Changes within groups were analyzed with paired test statistics (paired *t*-test or Wilcoxon matched-pairs signed-rank test).

## Results

Mean age of subjects was 53.6 ± 7.3 years, with a mean BMI of 25.3 ± 2.3 kg/m^2^. The mean blood pressure was 139 ± 6/85 ± 4 mmHg and the mean homocysteine level was13.61 ± 2.43 μmol/l. Detailed baseline characteristics are summarized in Table 1.

**Table 1:** Anthropometric data and baseline conditions at visit 1

	Total group (*n* = 25)	Men (*n* = 18)	Women (*n* = 7)
Age [years]	53.6 ± 7.3	51.6 ± 7.1 ^a^	58.9 ± 4.9 ^a^
BMI [kg/m^2^]	25.3 ± 2.3	25.49 ± 2.3	25.1 ± 2.4
HCY at screening [μmol/l]	13.61 ± 2.44	13.71 ± 2.49	13.37 ± 2.46
lnRHI at visit 1 [index]	0.57 ± 0.29	0.55 ± 0.27	0.63 ± 0.36
Systole at visit 1 [mmHg]	139.2 ± 5.7	140.1 ± 6.1	136.9 ± 3.8
Diastole at visit 1 [mmHg]	85.0 ± 3.9	84.6 ± 4.1	86.0 ± 3.2

Results are depicted for the whole study group, and separated by gender. ^a^ is indicating significant gender differences. Results are presented as mean ± standard deviation

**Fig. 1:**
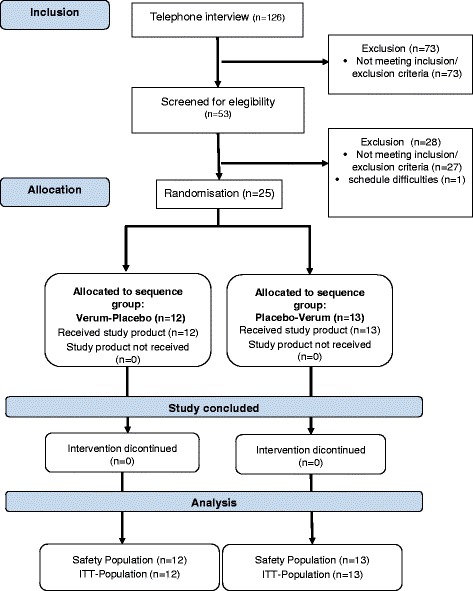
Disposition of subjects

Twenty-five subjects were randomized to the sequence groups and all subjects finished the study according to the protocol. Detailed subject allocation is shown in Fig. 1.

The primary endpoint, defined as change of endothelial function after 4 weeks supplementation with AbMIP showed significant (*p* = 0.047) difference when compared to placebo. *Cohens d* = 0.642 indicates a medium sized effect for endothelial function. Fasting endothelial function improved after 4 weeks supplementation with AbMIP. In contrast, endothelial function decreased under placebo supplementation (Table 2). After intake of ABMIP the postprandial reaction was more pronounced compared to placebo but without reaching a significant difference (Table 2). In both groups, a higher postprandial reaction after supplementation phase was observed. Systolic blood pressure decreased significantly under AbMIP from 138 ± 8 mmHg to 133 ± 9 mmHg (*p* = 0.002). However, due to a distinct placebo effect, statistical significance was not achieved (*p* = 0.586). Diastolic blood pressure also decreased under both study products but without significance within study groups (before and after supplementation) or between study groups. Homocysteine levels were significantly (*p* < 0.0001) lower after supplementation with AbMIP when compared to placebo (Table 3). AbMIP decreased elevated homocysteine levels in 68% of subjects to values below 10 μmol/l (17 out of 25 subjects). In contrast, under placebo only 4 subjects out of 25 (16%) reached values below 10 μmol/l.

**Table 2:** Effects of AbMIP and placebo on endothelial function, blood pressure and triglyceride

	Placebo			AbMIP			Product effect	
	Pre supplementation	Post supplementation	∆ post-pre supplementation	Pre supplementation	Post supplementation	∆ post-pre supplementation	*p*-value	*cohens d*
lnRHI [index]	0.603 ± 0.274 (0.49–0.716)	0.552 ± 0.274 (0.439–0.665)	−0.052 ± 0.271 (−0.163–0.06)	0.481 ± 0.373 (0.327–0.635)	0.551 ± 0.339 (0.411–0.691)	0.070 ± 0.327 (−0.065–0.205)	0.047	0.642
systole [mmHg]	136.8 ± 7.9 (133.5–140.0)	133.2 ± 6.9 (130.4–136.1)	−3.5 ± 6.1 (−6.0–(−1.0)) ^a^	137.4 ± 7.7 (134.2–140.6)	132.8 ± 9.1 (129.1–136.6)	−4.6 ± 6.6 (−7.3–(−1.8)) ^a^	0.586	0.221
diastole [mmHg]	84.8 ± 4.2 (83.1–86.6)	83.5 ± 4.4 (81.7–85.3)	−1.4 ± 4.3 (−3.1–0.4)	83.2 ± 4.5 (81.4–85.1)	82.2 ± 5.9 (79.8–84.6)	−1.0 ± 3.3 (−2.4–0.3)	0.763	0.119
TG [mg/dl]	116.2 ± 53.0 (94.4–138.1)	116.3 ± 43.0 (98.5–134.0)	0.0 ± 38.3 (−15.8–15.8)	117.0 ± 46.5 (97.8–136.2)	103.0 ± 40.4 (86.3–119.6)	−14.0 ± 27.9 (−25.5–(−2.5)) ^a^	0.108	0.533
	Pre fat meal	post fat meal	∆ post-pre fat meal	Pre fat meal	post fat meal	^∆^ post-pre fat meal		
lnRHI [index]	0.552 ± 0.274 (0.439–0.665)	0.754 ± 0.261 (0.646–0.862)	0.202 ± 0.234 (0.106–0.299)^∆^	0.551 ± 0.339 (0.411–0.691)	0.873 ± 0.171 (0.802–0.943)	0.322 ± 0.384 (0.164–0.48)^∆^	0.201	0.659

Effects of AbMIP and placebo on endothelial function, blood pressure and triglycerides before and after intervention and on postprandial endothelial function before and after high fat meal. Results are presented as mean ± standard deviation (95% CI). ^a^ is indicating significant post to pre supplementation changes within group and ^∆^ significant post to pre fat meal changes within group

**Table 3:** Effects of AbMIP and placebo on homocysteine (HCY), ADMA and HbA1c

	Placebo	AbMIP	Product effect	
	Post supplementation	Post supplementation	*p*-value	*cohens d*
HCY [μmol/l]	11.95 ± 1.85 (11.19–12.72)	9.10 ± 1.94 (8.3–9.9)	<0.0001	2.415
ADMA μmol/l]	0.632 ± 0.088 (0.56–0.69)	0.638 ± 0.107 (0.59–0.68)	0.649	0.185
HbA1c [%]	5.34 ± 0.38 (5.19–5.50)	5.37 ± 0.41 (5.20–5.54)	0.846	0.210

Results are presented as mean ± standard deviation (95% CI)

ADMA and HbA1c remained unchanged after supplementation with AbMIP and placebo. No significant differences were observed (Table 3). Compliance rate for both study products was above 85%. Tolerability of study products was very good. All except one subject assessed the tolerability of AbMIP as “good”. One subject chose the category “mildly unpleasant”, due to “possibly somewhat bloating”. No subject rated the tolerability as “very unpleasant” Routine blood parameters and adverse events did not indicate safety issues. No related adverse events occurred with AbMIP supplementation. Analysis of safety parameters revealed an additional beneficial effect of the study product: Triglyceride levels decreased significantly under AbMIP (*p* = 0.0166), but remained stable under placebo (*p* = 0.9959; Table 2). The difference between products did not reach statistical significance (*p* = 0.108). Noteworthy is one outlier in the placebo group with abnormal high values before supplementation which were completely different from all other time points, e.g., screening. After exclusion of this outlier, there was a significant difference (*p* = 0.026) in favour of AbMIP.

## Discussion

A current fact sheet of the WHO on CVDs states that most CVDs can be prevented by addressing behavioural risk factors such as tobacco use, unhealthy diet and obesity, physical inactivity and harmful use of alcohol using population-wide strategies [1]. The WHO also states that individuals with CVDs or individuals at high cardiovascular risk (due to the presence of one or more risk factors such as hypertension, diabetes, hyperlipidaemia or already established disease) need early detection and management [1]. However, some risk factors, like elevated blood pressure, mildly impaired endothelial function or moderately elevated homocysteine levels are first signs of vascular changes but are not always the reason for early treatment. First steps should be consequent lifestyle changes including healthier diet, increased physical activity, reduction of tobacco-use and alcohol-consumption [23]. Published studies demonstrate a beneficial effect of different nutritional ingredients on early symptoms of CVD, like endothelial dysfunction [5–10, 18–21]. AbMIP combines ingredients that influence different physiological processes and modify functions involved in the complex process of CVD development. Furthermore, it contains ingredients that help to cover an increased demand when in a diseased state (e.g., L-arginine and folic acid) [18, 24]. The current randomized, double-blind, placebo-controlled cross-over study evaluated the effects of AbMIP in subjects with early signs of CVDs (elevated blood pressure and hyperhomocysteinemia). Four weeks of supplementation with AbMIP led to a significant difference in fasting endothelial function (endothelium dependent vasodilation) and significantly lowered homocysteine levels in comparison to placebo supplementation.

Due to the multi-ingredient character of AbMIP, different mechanisms could be responsible for the observed effectiveness. Anti-inflammatory and anti-oxidant effects can be attributed to Pycnogenol®, for example [25]. Furthermore, there is evidence that Pycnogenol® and arginine stimulate NO production [26, 27]. Improvement of endothelium-dependent vasodilation has been demonstrated in vivo in patients with stable coronary disease for 200 mg Pycnogenol®/day [8], healthy subjects with 180 mg Pycnogenol®/day [7] and borderline hypertensive, hyperlipidemic and hyperglycaemic individuals with 150 mg Pycnogenol®/day [28]. In the current trial the study product contained 80 mg Pycnogenol® in combination with arginine, B-vitamins and further ingredients. The effects were assessed after 4 weeks of supplementation. Intake of the multi-ingredient product, AbMIP, led to significantly lower homocysteine levels compared to placebo. Likewise systolic blood pressure significantly decreased after supplementation with AbMIP, however, when compared with the placebo group the difference was not statistically significant due to strong placebo-effects. The difference of systolic blood pressure under placebo also decreased significantly within the group. It is known from literature, that placebo effects are quite commonly detected in hypertension trials [29], especially regarding systolic blood pressure [30, 29]. Consequently, the lack of efficacy for systolic blood pressure in comparison to placebo may be due to strong placebo effects and possibly due to the relatively short supplementation phase, which was adapted to the primary endpoint change of endothelial function. In order to mitigate any blood pressure placebo effects, it is supposed, that a longer supplementation phase would have been more suitable.

While significant effects of AbMIP on fasting endothelial function were observed, postprandial endothelial function did not reveal significant effects. Nevertheless the postprandial reaction after supplementation with AbMIP was more distinct when compared to placebo. In further studies, with the main emphasis on postprandial endothelial function, it would be more suitable to examine the effects of AbMIP before and after supplementation in larger sample sizes in order to prove effectiveness Another potential weakness of the study relates to different starting levels of the fasting endothelial function. Although nutrition, physical activity and lifestyle were controlled during the study period, subjects were instructed not to change their behavior and measurement conditions were highly standardized, the starting levels of fasting endothelial function were slightly different between placebo and AbMIP. These differences were not significant, however, this aspect needs to be considered for interpretation of results. Baseline time points were separated by a period of 3 months. It is known, that endothelial function is influenced by different factors, like temperature, season, hormonal status, psychological stress or nutritional status [31–33]. In this study, all controllable and influencing factors were considered (e.g., temperature control, acclimatisation time, female hormonal status, time of day). For determination of changes of fasting endothelial function, it is important to record individual changes and therefore include measurements before and after intervention. For this study a design with a period of 4 weeks of supplementation was chosen. This period is is supposed to be long enough to allow detection of possible effects but also short enough to minimize seasonal and other unpredictable influences. Although baseline levels were slightly different, individual changes demonstrated significant influences of AbMIP versus placebo. This indicates high potential of AbMIP for improvement of endothelial function in such a collective. Studies with larger sample sizes would be required to confirm the results.

During the 4-week supplementation phase of the current study, no safety concerns were noted for AbMIP. The tolerability was excellent and no related adverse events occurred. Altogether, AbMIP is a safe and well-tolerated preparation with high potential for the benefit of vascular health by improving endothelial function and lowering homocysteine levels. In the current trial, the effectiveness of AbMIP was evaluated in non-medicated subjects with elevated blood pressure and hyperhomocysteinemia. In future, it would be very interesting to investigate the influence of AbMIP on blood pressure after longer periods of supplementation.

## Conclusion

AbMIP, containing Pycnogenol®, arginine, folic acid, vitamins B6 and B12 as the main active ingredients, demonstrated significant improvement of fasting endothelial function and homocysteine levels in subjects with elevated blood pressure and hyperhomocysteinemia when compared against placebo control. Further research is needed to investigate the blood pressure lowering effects and confirm the effects shown in this study. AbMIP is very well tolerated and demonstrated high potential for vascular health due to relevant effects on endothelial function and hyperhomocysteinemia.

## Abbreviations

AbMIP: Arginine-based multi ingredient product
ADMA: Asymmetric dimethyl arginine
CVD: Cardiovascular diseases
ECG: Electrocardiography
EDTA: Ethylenediaminetetraacetate
GCP: Good clinical practice
HCY: Homocysteine
ICH: International conference on harmonisation
lnRHI: Natural log of reactive hyperemia Index
NO: Nitric oxide
PT: Prothrombin time
RHI: Reactive hyperemia Index
WHO: World Health Organisation
